# Building generic anatomical models using virtual model cutting and iterative registration

**DOI:** 10.1186/1471-2342-10-5

**Published:** 2010-02-08

**Authors:** Mei Xiao, Jung Soh, Oscar Meruvia-Pastor, Eric Schmidt, Benedikt Hallgrímsson, Christoph W Sensen

**Affiliations:** 1 Sun Center of Excellence for Visual Genomics, Department of Biochemistry and Molecular Biology, Faculty of Medicine, University of Calgary, 3330 Hospital Drive NW, Calgary, T2N 4N1, Canada; 2 Morphometrics Laboratory, Department of Cell Biology and Anatomy, Faculty of Medicine, University of Calgary, 3330 Hospital Drive NW, Calgary, T2N 4N1, Canada

## Abstract

**Background:**

Using 3D generic models to statistically analyze trends in biological structure changes is an important tool in morphometrics research. Therefore, 3D generic models built for a range of populations are in high demand. However, due to the complexity of biological structures and the limited views of them that medical images can offer, it is still an exceptionally difficult task to quickly and accurately create 3D generic models (a model is a 3D graphical representation of a biological structure) based on medical image stacks (a stack is an ordered collection of 2D images). We show that the creation of a generic model that captures spatial information exploitable in statistical analyses is facilitated by coupling our generalized segmentation method to existing automatic image registration algorithms.

**Methods:**

The method of creating generic 3D models consists of the following processing steps: (i) scanning subjects to obtain image stacks; (ii) creating individual 3D models from the stacks; (iii) interactively extracting sub-volume by cutting each model to generate the sub-model of interest; (iv) creating image stacks that contain only the information pertaining to the sub-models; (v) iteratively registering the corresponding new 2D image stacks; (vi) averaging the newly created sub-models based on intensity to produce the generic model from all the individual sub-models.

**Results:**

After several registration procedures are applied to the image stacks, we can create averaged image stacks with sharp boundaries. The averaged 3D model created from those image stacks is very close to the average representation of the population. The image registration time varies depending on the image size and the desired accuracy of the registration. Both volumetric data and surface model for the generic 3D model are created at the final step.

**Conclusions:**

Our method is very flexible and easy to use such that anyone can use image stacks to create models and retrieve a sub-region from it at their ease. Java-based implementation allows our method to be used on various visualization systems including personal computers, workstations, computers equipped with stereo displays, and even virtual reality rooms such as the CAVE Automated Virtual Environment. The technique allows biologists to build generic 3D models of their interest quickly and accurately.

## Background

Spatial information of biological structures has been used to analyze their functions and to relate their shape changes to various genetic parameters [1-4]. In particular, using 3D generic models to statistically analyze trends in biological structure changes is an important tool in morphometrics research [1,2,4-10]. In order to be suitable for statistical analysis, a generic 3D model must be a single averaged model representing all individual 3D models in the same population of a study [5,11]. An averaged 3D model is a commonly used form of a generic 3D model. The creation of an averaged model captures information that can be exploited in statistical analysis of real populations. By comparing averaged models and dispersion around them, anatomical differences can be quantified across groups that differ in some underlying causal or exploratory factors, such as genetics, gender, and drug treatment [3]. The comparisons can be made between 'static' morphological states, where the subjects for comparison are at the same developmental state or they can be between 'dynamic' states, where comparisons are made between various stages of the subject's growth. Therefore, a technique for creating high throughput 3D generic models is needed to collect and manage large numbers of subjects quickly and efficiently. Such a technique will enable researchers to discover a wide range of traits to their interest in both natural and clinical settings. Generic 3D models can also be used in automatic segmentation [1], medical education, virtual crash testing, therapy planning and customizing replacement body parts [11,12]. Hence, in medical and biological studies, 3D generic models built for a range of populations are in high demand.

In order to create valid 3D generic models from 2D image stacks, more attention should be paid to two essential steps - image segmentation and image registration. Image registration is the process to find a 3D transformation that can map the same anatomical region from one subject into another one. This process is essential in clinical and research applications because researchers often need to compare the same anatomical region scanned using different modalities or at different time points [13]. Image segmentation is needed when we try to retrieve the spatial information of certain biological structures after applying in vivo imaging technologies such as MRI. This step is generally indispensable because 3D image stacks generated from in-vivo scanners usually contain a large amount of superfluous information that is irrelevant to immediate diagnostic or therapeutic needs.

With the tremendous advancements in medical imaging technologies such as CT, PET, MRI, and fMRI, we are now able to capture images of biological structures and their functions more clearly than ever before. Additionally, advanced technologies from other fields such as computer vision, computer graphics, image processing and artificial intelligence have been used to analyze 2D medical images of various modalities [1]. However, due to the complexity of biological structures and their shape information overlaying on medical images, it is still an exceptionally difficult task to quickly and accurately create 3D generic models for a population of a study.

Due to the difficulties with automating the segmentation task, enhanced manual segmentation software is still widely used. Various image processing algorithms have been produced to minimize user interactions and increase segmentation accuracy [14]. However, the current enhanced manual segmentation approaches are still quite laborious; many times it requires a well-trained user to interact with every 2D image slice. Therefore, in order to achieve accurate 3D reconstruction of a region, structure, or tissue of interest [6], it is necessary to entail specifically tailored solutions that combine and integrate different 3D segmentation algorithms [15] that may still necessitate manual segmentation on each 2D image slice. To redress such persistent drawbacks, we have developed a generalized virtual dissection-based method for creating generic models. In comparison to our previous virtual dissection technique [16], the method now allows user-define curves for indicating cutting surfaces and employs enhanced iterative registration to better handle shape variations. In addition, the resulting software is now publicly available. We show that the creation of an averaged model that captures spatial information exploitable in statistical analyses of organ shape is facilitated by coupling our generalized segmentation method with existing automatic image registration algorithms [13].

## Methods

### Materials

**Figure 1:**
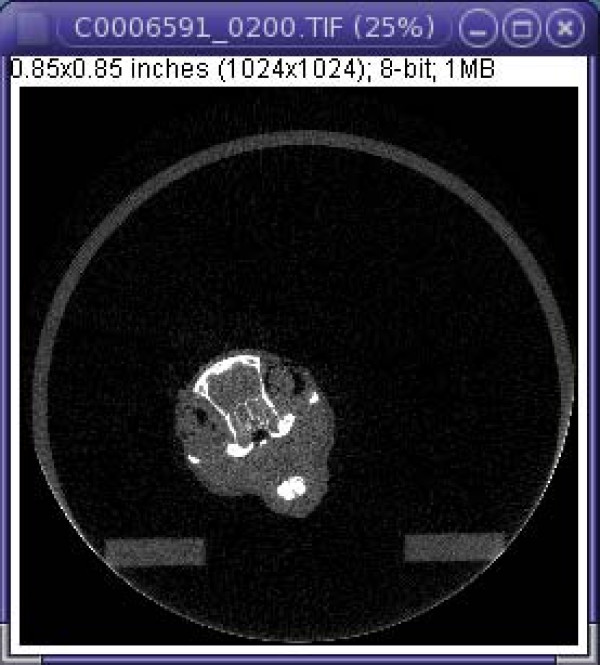
**A slice of a 2D image stack obtained from a whole body scan**.

2D image stacks of mice whole-body micro-computed tomography (μ-CT) scans were provided by the Morphometrics Laboratory at the University of Calgary. Eight male and eight female laboratory mice from the same strain (AWS) were scanned. The female mice were 54 to 61 days old and weighed 16 to 21 grams; the male mice were 61 days old and weighed 20 to 25 grams. All individuals were scanned at a resolution of 35 μm. Each slice of the volumetric dataset is 1024 × 1024 pixels and the intensity of each pixel ranges from 0 to 255 (Figure 1). The total number of images in a stack ranges from 2100 to 2400. The process of creating generic 3D models is illustrated by describing the process of creating the 3D generic left mandible model using our method. It should be noted, however, that the left mandible was picked solely for the purpose of illustration and our method can be used for creating a 3D generic model of other anatomical structures as well.

### Overview of the method

The method pipeline contains the following major steps: (i) scanning subjects to obtain image stacks; (ii) creating individual 3D models from the stacks; (iii) cutting each model to generate a sub-model of the user's interest; (iv) making image stacks that contain only the information pertaining to the sub-models; (v) iteratively registering the corresponding new 2D image stacks from the previous step; (vi) averaging the newly created sub-models based on intensity to produce the generic model from all the individual sub-models. All the algorithms are implemented using Java and C++ based on functionalities from open source toolkits VTK (Visualization Toolkit [17]), ITK (Insight Segmentation and Registration Toolkit [13]) and ImageJ [18]. Both volumetric data and surface model for the generic 3D model are created at the final step.

### 3D model reconstruction

**Figure 2:**
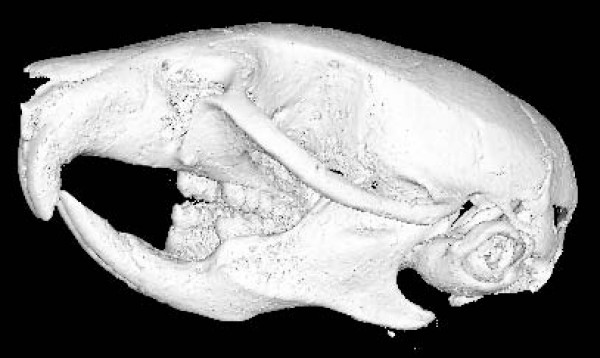
**Reconstructed 3D mouse skull model**.

Since the imaging data we have are mice whole-body scans, the information of all the biological structures are contained in the image stacks. The sub-model of our interest here is the left mandible. Instead of separating the data for the left mandible from each image, we reconstruct the skull (Figure 2) of each mouse using the Marching Cubes algorithm in VTK based on the pixel intensity of the bone structure.

### Sub-model of interest creation

Our reconstructed 3D model is a representation of the whole mouse skull. In order to retrieve the sub-model, our custom-developed cutting tools are used to cut the 3D skull model until the desired separation of the sub-model is achieved.

**Figure 3:**
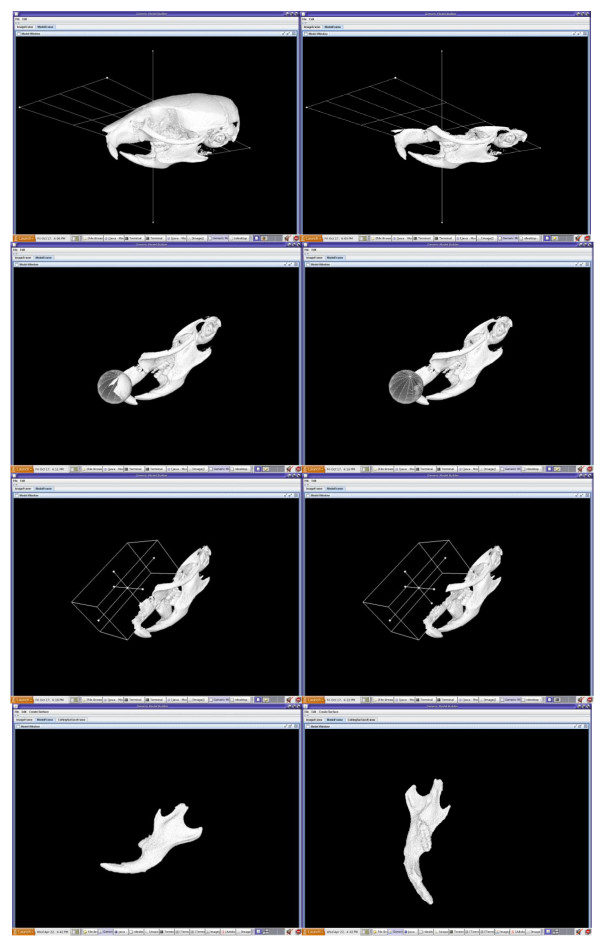
**Using various cutting tools to produce a desired sub-model (left mandible)**.

Our cutting instruments can be a plane, ball, box, or user-defined curve. The planes, balls and boxes are all virtual models that can be manipulated interactively by using the computer mouse. As illustrated in Figure 3, the plane can be rotated, zoomed in and out, and translated, while the arrow shows the normal of the plane. Therefore users can decide where to set the plane to remove any portion that is of no interest to them. The ball and the box can also be rotated, scaled and translated using the computer mouse to remove the parts that are of no interest to the users.

**Figure 4:**
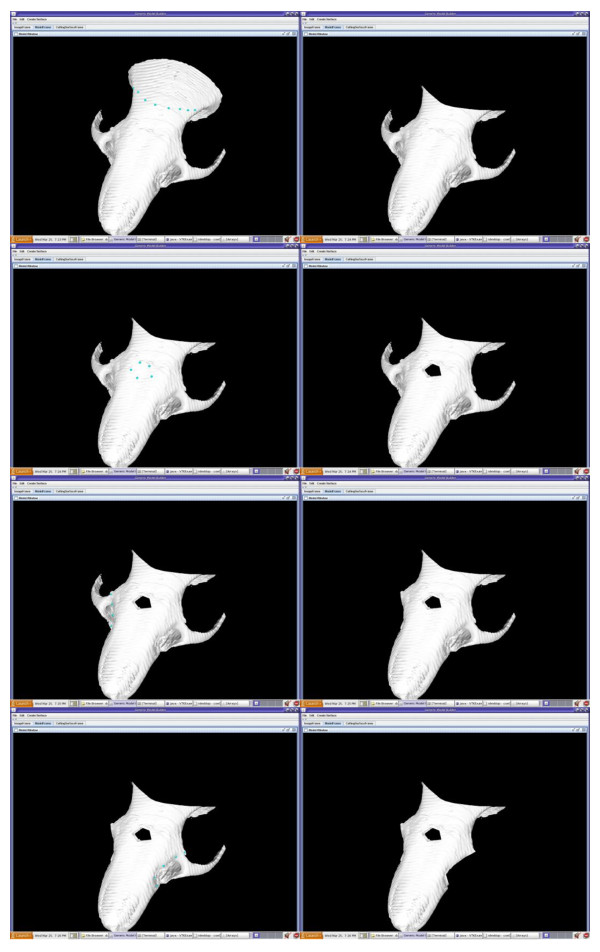
**User defined cutting curve**. Users can choose to remove irregular sections from the model by using a series of dots to indicate the intended cutting curve.

Users can also simulate a cutting curve by putting a series of dots on the model through computer mouse double clicks, as Figure 4 shows. Users can manipulate the model by rotating, translating, or zooming in or out to observe the area that they are interested in. The order in which the dots are placed is significant as they are used as the data points for interpolating a best-fitting curve. If the dots are put in counterclockwise order, the part of the model that is above or to the left of the simulated curve is removed; otherwise the part below or to the right is removed. If a closed curve is simulated, the portion enclosed by the closed curve is removed. The cutting tools are implemented using functionalities from VTK.

### Creating corresponding 2D image portions of the sub-model

**Figure 5:**
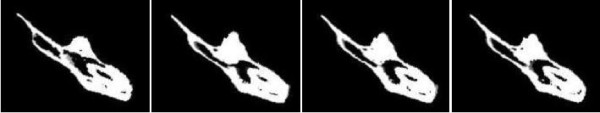
**Updated 2D image stack**. Part of an updated 2D image stack showing slices 160, 170, 180, and 190, respectively (from left to right). After the cutting process, 2D image stacks are updated using the information on the cutting tools used. 2D image stacks that contain only information about the sub-model of interest are created automatically.

While the users are cutting the model, all the cuts are recorded and the coordinates used by the cutting tools such as the plane's center and normal, the sphere's center ad radius, the planes that composed the box, and the dots in the user-defined cutting curve are recorded into a text file. After the cutting process is finished, the intensities of the pixels in the image stack are updated according to the cutting information. Intensities of pixels that correspond to the model stay the same and the rest are set to 0. After this process is finished, we obtain a new image stack that contains only the data for the sub-model. The above steps are repeated to process all the mice image stacks to create the sub-models and the new 2D image stacks. The resulting 2D image stacks that contain only the sub-model information (see Figure 5) are registered and the generic model for the sub-model (the left mandible) is created. The production and averaging of 2D image portions are performed using functionalities in ImageJ.

### Iterative image registration

The following registration algorithms are used.

1. *Rigid 3D image registration*. In order to align the entire set of sub-models into the same space automatically, an intensity-based rigid 3D registration algorithm which uses a mean square metric, a linear interpolator, a versor rigid 3D transform and a versor rigid 3D transform optimizer inside ITK is used to register the images.

2. *Affine 3D image registration*. Due to the variations of each individual sub-model, rigid 3D image registration creates local misalignments and the averaged model created based on only rigid image registration might not be an average representative. Therefore, affine 3D image registration is also available in our package to further align the models. An intensity-based affine 3D registration algorithm which uses a mean square metric, a linear interpolator, an affine transform and a regular step gradient descent optimizer inside ITK is applied for affine registration.

3. *Non-rigid (deformable) image registration*. The global affine transformation from the previous step might create some remaining local shape variations. Therefore, in order to sharpen the blurry average images, a non-rigid image registration can also be used after step 2. An intensity-based deformable 3D registration algorithm which uses a mean square metric, a linear interpolator, a B-spline based transform and a LBFGS (limited memory Broyden-Fletcher-Goldfarb-Shanno update) optimizer inside ITK is applied for further deformable image registration.

**Figure 6:**
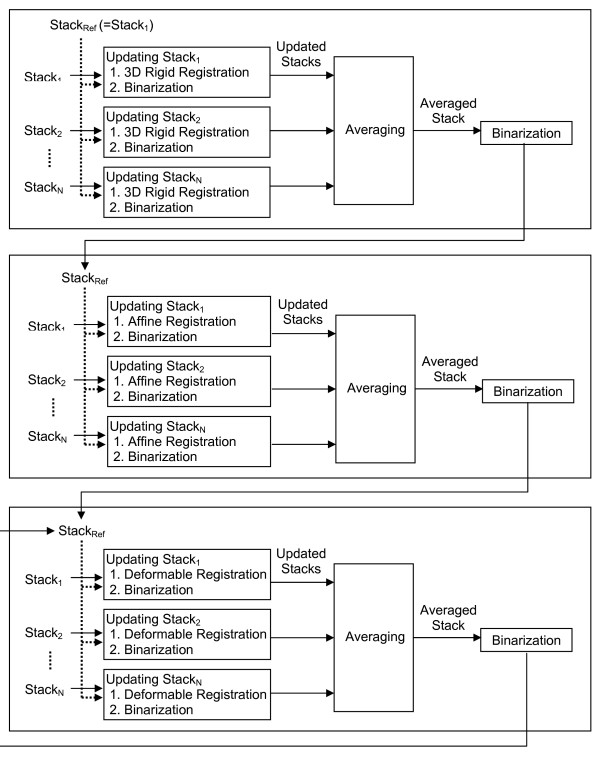
**Iterative image registration**. The reference stack is iteratively refined by performing a series of 3D registration algorithms on each stack: rigid 3D image registration, affine 3D image registration, and non-rigid deformable 3D image registration. The non-rigid registration step can be repeated to achieve more accurate registration.

We use a similar iterative image registration protocol to the one mentioned in [6] (see Figure 6 for a flow chart of the process).

1. We randomly pick a subject from the female group as a reference and register every image stack to this reference stack using 3D rigid registration. After each registration step, the intensities of the images are turned into binary such that pixels with intensities 255 belong to the model and pixels with 0 belong to the background. Then we average corresponding pixel intensities from all the stacks to create the averaged image stack [19]. The same registration process is applied to the male group.

2. Averaged models are created from the previous step by using the global median of the pixel intensities as the threshold value for binarizing the averaged image stack. An affine transformation based image registration is applied again to all the images that have been processed by rigid transformation from the previous step in the same way as described in the previous step and new averaged image stacks are created.

3. The previous step is repeated, but this time B-Spline based deformable image registration is applied to all the images that have been processed by affine transformation from the previous group.

4. The previous step can be applied repeatedly to all the images that have been processed by deformable transformation from the previous group in order to achieve more accurate registrations.

### Intensity based image averaging

After the iterative image registration step, all image stacks of the sub-models (the left mandibles) are registered. At this point, we can use intensity based image averaging technique as described in [19].

The global median of the averaged image intensities is used to apply the marching cube algorithm to the averaged image stacks [19] to extract the generic left mandible model that represents the average shape of all the left mandibles across all the subjects in the same population.

## Results

### Generic model building

We have developed a generalized virtual dissection-based method for the creation of generic models from 2D image stacks of a group of individuals. To illustrate our novel generic models creation technique, whole body scans of eight female mice and eight male mice are used to create averaged 3D models of the left mandible. For each subject, the left mandible 3D model is created using our cutting tools and the corresponding 2D image stack that contains only information of the left mandible is also generated.

### Validation of the iterative registration

Once we have 16 left mandible models, we register the image stacks for both male and female mice. Corresponding pixels in the images of the female/male group are averaged to create an averaged image stack. Within the averaged image stack, the blurry image areas result from the misaligned sections. Therefore, the sharper the averaged images are, the better the registration process is. We use the ratio of the number of pixels with intensity 255 to the number of pixels with non-zero intensity to measure the performance of the registration process (see Table 1). The bigger the ratio is, the better the models are aligned.

**Table 1:** Comparison of image registration accuracy

		No. of pixels with intensity 255/No. of pixels with non-zero intensity after registration		
		
Mouse Group	Averaged model	Versor based 3D rigid registration	Affine transformation based 3D registration	B-Spline deformable transformation based 3D registration
Female Group	F2 as reference	0.4774	0.5732	0.6241
	
	F3 as reference	0.4819	0.5761	0.6470
	
	F4 as reference	0.4986	0.5842	0.6658
	
	F5 as reference	0.4836	0.5723	0.6458
	
	F6 as reference	0.4478	0.5499	0.6598
	
	F7 as reference	0.4791	0.5570	0.6307
	
	F8 as reference	0.4781	0.5618	0.6406
	
	F9 as reference	0.4861	0.5988	0.6546

Male Group	M2 as reference	0.5534	0.5954	0.6219
	
	M3 as reference	0.5300	0.5904	0.6218
	
	M4 as reference	0.5350	0.5871	0.6593
	
	M5 as reference	0.5400	0.5939	0.6452
	
	M6 as reference	0.5286	0.5844	0.6326
	
	M7 as reference	0.5380	0.5899	0.6347
	
	M8 as reference	0.5285	0.5960	0.6323
	
	M9 as reference	0.5332	0.5912	0.6365

**Figure 7:**
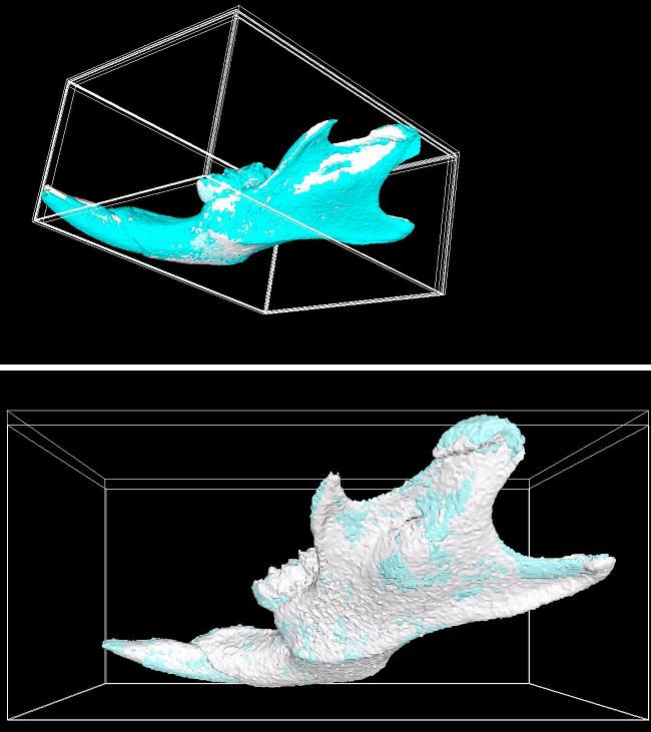
**Misalignments after 3D rigid registration and affine registration**. Two models shown in different colors (gray and cyan) are superimposed. On the top, after 3D rigid registration, there are obvious misalignments on the front of the mandible and towards the back of the mandible. On the bottom, after the affine registration, there are fewer misaligned areas.

As illustrated in Figure 7, if only 3D rigid registration is applied, we can clearly observe misaligned areas. Once an affine transformation based registration is applied, less misaligned areas can be identified. From the ratios that are listed in Table 1, we can conclude that, after several registration procedures are applied to the image stacks, we can create averaged image stacks with sharp boundaries.

If we choose different initial reference subjects, will the averaged models be very different? We test the effect by choosing different subjects as the initial reference subjects to create the averaged models. We generate multiple averaged models, each using a different initial stack as the reference stack. For example, in Table 2, "Average F2" means d female model using female number 2 (F2) as the reference. Afterthe average producing the different averaged models, we register all of them with respect to a neutral averaged model to make their comparison meaningful and to avoid any potential bias. We used one male averaged model to register all the female group averaged models. Similarly, we registered all the male group averaged models with one female averaged model.

**Table 2:** Dice index to evaluate the similarities between two averaged models created from different initial references

		Average F2	Average F3	Average F4	Average F5	Average F6	Average F7	Average F8	Average F9
Female Group	Average F2	1	0.9768	0.9745	0.9795	0.9767	0.9786	0.9775	0.9753
	
	Average F3		1	0.9768	0.9776	0.9760	0.9762	0.9770	0.9757
	
	Average F4			1	0.9747	0.9745	0.9744	0.9757	0.9742
	
	Average F5				1	0.9770	0.9789	0.9779	0.9759
	
	Average F6					1	0.9782	0.9774	0.9748
	
	Average F7						1	0.9779	0.9748
	
	Average F8							1	0.9765
	
	Average F9								1

		**Average M2**	**Average M3**	**Average M4**	**Average M5**	**Average M6**	**Average M7**	**Average M8**	**Average M9**
	
Male Group	Average M2	1	0.9802	0.9776	0.9780	0.9784	0.9785	0.9800	0.9787
	
	Average M3		1	0.9785	0.9792	0.9791	0.9787	0.9796	0.9796
	
	Average M4			1	0.9794	0.9791	0.9785	0.9789	0.9781
	
	Average M5				1	0.9790	0.9787	0.9793	0.9789
	
	Average M6					1	0.9792	0.9796	0.9776
	
	Average M7						1	0.9800	0.9778
	
	Average M8							1	0.9790
	
	Average M9								1

Dice index measurement [20] is used to evaluate the similarities between averaged models starting from different reference subjects, after the additional registration procedure to facilitate direct comparison. As shown from Table 2, the similarity measures are from 0.97 to 0.98 among different averaged models. We believe that the rest 0.02 to 0.03 differences are due to the system error caused by the registration process. For the female mice group, the mean dice index is 0.976464, the standard deviation is 0.001489 and the coefficient of variation is 0.001524. For the male mice group, the mean dice index is 0.9789, the standard deviation is 0.000698 and the coefficient of variation is 0.000713. Therefore, we can see that in this case, starting from different reference subject will not affect the averaged models.

Brandt et al. [6] tested the honeybee brain average shape property. They used the residual non-rigid deformation necessary to map the subjects' coordinate another after they have been normalized with respect to position and size. They found out that the averaged honeybee brain model using the iterative registration method is indeed a reasonable approximation of a shape centroid of the population. We measure the RMSE (root mean square error) of voxels between every two models and between every model and the averaged model. As shown from Table 3, the RMSE between every model and the averaged model is smaller than the RMSE between that model and every other model. Combining our RMSE computation and the test from Brandt et al. [6], we believe using the iterative registration algorithm [6] will give us a practical average model that captures the spatial information of the population. Our method is very flexible and easy to use such that anyone can use image stacks to create models and retrieve a sub-region from it at their ease. The image registration time varies depending on the image size and the desired accuracy of the registration.

**Table 3:** Root mean square error (RMSE) between models

		F2	F3	F4	F5	F6	F7	F8	F9	Averaged Model
Female Group	F2	0	16.93	17.82	18.21	19.08	18.75	19.52	19.70	14.99
	
	F3		0	17.54	17.52	17.74	18.29	18.58	18.78	15.05
	
	F4			0	18.01	19.44	17.97	19.06	18.84	16.32
	
	F5				0	17.87	16.58	18.05	15.92	15.51
	
	F6					0	18.20	17.83	19.32	16.10
	
	F7						0	18.18	16.99	15.47
	
	F8							0	18.76	16.29
	
	F9								0	17.02

		**M2**	**M3**	**M4**	**M5**	**M6**	**M7**	**M8**	**M9**	**Averaged Model**
	
Male Group	M2	0	16.62	17.00	17.20	17.68	16.68	16.97	16.80	13.89
	
	M3		0	16.10	15.62	16.33	16.01	16.36	15.42	12.96
	
	M4			0	17.59	16.40	17.17	16.39	17.36	14.56
	
	M5				0	18.34	17.08	16.35	15.78	14.50
	
	M6					0	16.45	17.73	17.56	14.85
	
	M7						0	17.37	15.95	13.41
	
	M8							0	17.11	14.21
	
	M9								0	13.71

### Binarization problem

Many studies considered complicated organs such as brain [4,9,10,21,22]. Inside the brain, different sub regions need to be considered during the registration process. Therefore, if one uniform intensity value is used to represent the organ, homogenous tissue mapping might not be available. However, in our study we would like to consider the organs with homogeneous intensities and structures. Therefore, we can use only one intensity value to represent the model and use it for registration and model averaging. This would reduce the registration time and increase the registration accuracy.

## Discussion

### Flexible module-based implementation

Our method is composed of five modules: 3D model reconstruction, sub-model of interest creation, production of 2D image stacks corresponding to the sub-models, image registration, and generic 3D model creation. Each module in this framework has various algorithms that can be applied according to the requirements of a specific scientific study.

For 3D model reconstruction from 2D image stacks, the marching cubes algorithm is the most popular one. Moreover, other reconstruction algorithms have been developed to improve the quality of the contour geometry [23,24]. Therefore, depending on the application requirements, different reconstruction algorithms can be used in our method to create polygonal models. Our cutting tools can be used to process polygonal models created from any reconstruction algorithm.

### Efficiency of the cutting approach

In order to automatically or semi-automatically create generic 3D models, different approaches have been proposed. However, those generic model building tools either need perfect individual models [5] or require costly human-computer interactions to retrieve 3D models. In [6], a brain atlas of the honeybee was constructed. The brain structures of the honeybee, such as neuropils and neurons, were manually segmented and labeled. Even with sophisticated algorithms [13] to help users to trace regions slice-by-slice quickly and accurately, manually processing thousands of images is still very labor intensive. Therefore, we focused on processing more slices with fewer human-computer interactions. Using a plane to separate a 3D polygon mesh has been used to refine a model created from CT or MRI image stacks [14]. Our approach can use not only a plane but also a box, a sphere, or even a user-defined curve to cut 3D models. More cutting algorithms can be added as well to quickly remove the portion that is of no interest to the users. Hence, with the cutting information, corresponding 2D image stacks can be updated automatically. Our approach can be used to create the desired models very quickly and automatically register images. Therefore, our method significantly shortens the generic model building time.

We used a Windows PC with dual CPUs to create all the left mandible models. The machine has two 2 GHz CPU with 2 GB memory. In order to retrieve one left mandible model, we need to process an image stack of size 1024 × 1024 × 500. The current machine setup cannot process this image stack at one time; therefore, we process the image stack in three consecutive parts. On the average we use 16.28 minutes and 14.75 cuts to retrieve a complete left mandible for the female mice group, and 16.2 minutes and 19.25 cuts for the male mice group (see Table 4). These times include both the waiting for the rendering time and the cutting manipulation time. On the average, it takes 3.31 minutes to render the female mouse model and 3.98 minutes to render the male mouse model initially.

**Table 4:** Processing time for model making

	Female mice	Male mice
Stack size	Image size: 1024 × 1024 Number of images: 500	Image size: 1024 × 1024 Number of images: 500

Average time to create a sub-model from a stack	16.28 minutes	16.2 minutes

Average number of cuts performed	14.75 cuts	19.25 cuts

### Image registration

Since image registration is an essential step towards creating generic models, numerous techniques have been developed to register corresponding 2D image stacks or 3D models. For some applications, averaged models created from the rigid registration step satisfy the requirements. For example, in [19], an intensity-based rigid image registration algorithm is applied to create a generalized shape image (GSI) which represents average values of the corresponding pixel intensities across all the image stacks. Even though this method yields some shape variations and not well-registered images create local differences from averaged images by using the gold standard (e.g. landmark based Procrustes average) it still can be used as a screening tool for the initial shape analysis. In [6] iterative averaging is used to register all the original images to the same reference to create an average, and then iteratively re-register the original images to the new average. Affine and non-rigid image registrations are applied in the honeybee brain atlas creation. A subsequent affine registration step removes more misaligned shape differences than applying only the rigid registration and creates a sharper averaged image, but relative shape differences might still remain. Nevertheless, compared with automatic deformable registration, affine registration requires fewer parameters and the computation time is relatively short. Therefore, depending on the requirements of the application, deformable registration can be used repeatedly to further remove the misalignments and create still sharper averaged images.

If the user wants to create an averaged surface model that is more like the gold standard Procrustes averaged model, a method for jointly registering and averaging 3D surface models, such as the one described in [5], can be used. Anatomical structures are modeled using a quadrangular mesh. The contour in each image slice is detected and then re-sampled using the same number of points. Then a permutation of points on each contour is performed to guarantee that every point in each model corresponds to the same anatomical region of the point with the same index in all other models. The points are finally averaged to create the generic model. The points are indexed on two integer coordinates, one of which represents the ordering of the initial image stacks. However, in order to use this approach, we have to pay attention to the alignment in the direction of slice ordering, since that method assumes that the anatomical structures along this direction are aligned automatically by the scanner. Therefore, rigid, affine or deformable registrations should still be used first to ensure that the anatomical structures along the direction are aligned. Subsequently, the multiple 3D anatomical surface models averaging algorithm [5] can be used to create an averaged surface model. Our package does not provide the quadrangular mesh building algorithm as described in [5], however our registration programs can still be used to align the anatomical structures along the slice ordering direction.

### Information on shape variation

The rigid, affine and non-rigid registration algorithms that we employ allow us to align all the subjects virtually and create the averaged models. Besides having the final averaged 3D models, all the transformations applied during the registration step are also available for visualizing shape changes and numerical morphometrical analysis such as global and local shape comparisons, strain tensor analysis, and modes of variations analysis [3,6,25]. The transformations are all available through ITK [13].

Versor based 3D rigid transformation has six parameters that represent a 3D rotation and a 3D translation. The rotation is specified by a versor quaternion and the translation is represented by a vector. The first three parameters define the versor and the last three parameters represent the translation in each dimension. Those parameters are available for further image analysis. A versor is defined as the quotient between two non-parallel vectors of equal length. Versors represent an orientation change in a vector, and they are a natural representation for rotations in 3D space [13].

In the above equation, *V *is a versor. *X *is a point in the 3D space. *C *is a vector that represents the rigid transformation center. The application of the versor onto the vector (X-C) is different from the regular vector product. However, in ITK, we can convert the versor product into the Euclidean matrix format. The 3D rotation matrix and the translation vector can be calculated from the versor product and can be saved for further analysis.

3D affine transformation can be represented as:

where *X *is a vector and represents a point in the 3D space, *A *is a 3 × 3 matrix and represents the affine transformation matrix, *C *is a vector and represents the transformation center, and *T *is a vector and represents the 3D translation. *X*' is the new position for *X *after the affine transformation. The affine registration from ITK that we utilized consists of rotation, scaling, shearing and translation in the 3D dimension. There are (3+1) × 3 parameters in this transformation. The first 3 × 3 parameters define A, the last 3 parameters define the translations for each dimension. The center of the transformation is automatically calculated from the programs and is also available.

B-Spline based non-rigid transformation [3,6,9,13] will generate a dense deformation field where a deformation vector is assigned to every point in the 3D space. The deformation field is available and can be saved in the form of a vector image from ITK. The deformation vector can be used to further analyze the local shape variations.

### Applicability of the method

Using our cutting tools to build models from 2D image stacks allows beginners in medical fields to learn anatomy intuitively and enjoy the process of separating the biological structures from the virtual body model before dealing with real subjects. Quickly and accurately creating various 3D averaged models can satisfy the requirements for a large number of models in virtual crash testing, therapy planning, and customizing replacement body parts. Large scale morphological studies that require quantification of anatomical features can be really tedious and might be very detailed and only focused on a few important measurements. Our method facilitates morphological studies by allowing anatomical structures to be measured and compared rapidly and in more detail. These tools help put morphological analysis at a similar level to other studies such as genetic and molecular studies where a large amount of data and measurements can be dealt with relatively quickly.

The issue of homology, which refers to biological structures that have the same function, is also addressed through our method. If we measure an average and do quantitative comparisons, we would want to compare the same anatomical region. This requires the two models being compared to be first registered correctly with each other, such that if one area of interest is picked in one model, it refers to the same region in the other model. The iterative registration employed in our approach can, to a large extent, reduce the misalignments. The method we developed optimizes the functionalities and technologies of existing toolkits and the resulting software package allows biologists to build their generic models more quickly and accurately.

As our virtual dissection tools are implemented in Java, they can run on both regular display systems and on the state-of-the-art CAVE Automated Virtual Environment [26], which is a 3D stereo-based 4-wall display system installed at the University of Calgary to provide users with a virtual immersive environment. One of the advantages of using this virtual reality system as a platform for our cutting tools is that users can treat both real world objects and virtual world objects quite the same way, which is not possible in a desktop computing environment or even in a single-wall stereo display environment. For example, users can move around in the display environment and view virtual objects from the "inside" such that the details operations can be easily understood. By harnessing the power of the CAVE and our cutting tools, users will have more flexibility including a wide variety of viewing perspectives and a high degree of freedom to set locations and orientations of the cutting tools. This is a definite advantage over ordinary desktop computing environments where the objects need to be frequently rotated to perceive their 3D structures.

## Conclusions

We have developed a new technique that uses virtual model cutting and iterative image registration to create generic models from 2D image stacks of a group of individuals. Our system allows biologists to build generic 3D models quickly and accurately. However, particularly complicated morphological structures, such as highly branched and convoluted designs that typify vascular or nervous networks, still pose a challenge to our generalized and enhanced method toward generic model creation. It is difficult to use the current manual virtual dissection tools to remove such sub-models from initial, unprocessed scans. More convenient and intuitive manual virtual dissection methods will be developed in our future research. Producing deformable models based on the current tools will also be an area of further development. Those deformable averaged models can then be used for automatically segmenting the anatomical structures. More advanced automated segmentation algorithms that utilize generic models will be studied to enable higher throughput analyses of anatomical structures in both medical and more general biological contexts. Quantification of 3D shape variations will also be studied based on our generic model building technique.

## Software availability and requirements

The implementation of our method is available for free downloading at http://www.visualgenomics.ca/~mxiao/research.html. The current version of the software has been tested on Unix Solaris 10 and Windows XP with .NET Framework 3.5. In order to run the program from our jar files, at least Java 1.6 need to be installed. ImageJ as well as shared (dynamically linked) libraries of VTK and ITK should also be installed.

Detailed installation and the user's guide are also available on the project website. VTK, ITK and ImageJ are all open source and freely available software toolkits.

## Competing interests

The authors declare that they have no competing interests.

## Authors' contributions

MX, JS, OEMP, EJS, BH and CWS participated in writing the manuscript and designing the technique. MX developed the computing framework. BH and CWS directed the research. All authors read and approved the final version of the manuscript.

## Pre-publication history

The pre-publication history for this paper can be accessed here:

http://www.biomedcentral.com/1471-2342/10/5/prepub

## References

[B1] Thompson PM Mega MS Narr KL Sowell ER Blanton RE Toga AW Sonka M, Fitzpatrick JM Brain image analysis and atlas construction Handbook of Medical Imaging: Medical Image Processing and Analysis 2000 2 SPIE Press 1063 1119

[B2] Small CG The Statistical Theory of Shape 1996 New York: Springer

[B3] Olafsdottir H Darvann TA Hermann NV Oubel E Ersboll BK Fangi AF Larsen P Perlyn CA Morriss-Key GM Kreiborg S Computational mouse atlases and their application to automatic assessment of craniofacial dysmorphology caused by the crouzon mutation Fgfr2C342Y Journal of Anatomy 2007 211 37 52 10.1111/j.1469-7580.2007.00751.x 17553099 PMC2375796

[B4] Barratt DC Chan CSK Edwards PJ Penney GP Slomczykowski M Carer TJ Hawkes DJ Instantiation and registration of statistical shape models of the femur and pelvis using 3D ultrasound imaging Medical Image Analysis 2008 12 258 374 10.1016/j.media.2007.12.006 18313973

[B5] Maschino E Maurin Y Andrey P Joint registration and averaging of multiple 3D anatomical surface models Computer Vision and Image Understanding 2006 1 16 30 10.1016/j.cviu.2005.06.004

[B6] Brandt R Rohlfing T Rybak J Krofczik S Maye A Westerhoff M Hege HC Menzel R Three-dimensional average-shape atlas of the honeybee brain and its applications The Journal of Comparative Neurology 2005 492 1 19 10.1002/cne.20644 16175557

[B7] Avants B Gee JC Shape averaging with differmorphic flows for atlas creation Proceedings of the IEEE International Symposium on Biomedical Imaging, 1: April 2004 2004 Arlington, VA 595 598

[B8] Argall BD Saad ZS Beauchamp MS Simplified intersubject averaging on the cortical surface using SUMA Human Brain Mapping 2006 27 14 27 10.1002/hbm.20158 16035046 PMC6871368

[B9] Ruckert D Frangi AF Schnabel JA Niessen WJ, Viergever MA Automatic construction of 3D statistical deformation models using non-rigid registration Lecture Notes in Computer Science: Medical Image Computing and Computer-Assisted Intervention-MICCAI 2001 2001 2208 Berlin Heidelberg: Springer 77 84

[B10] Rajamani KT Styner MA Talib H Zheng G Nolte LP Ballester MAG Statistical deformable bone models for robust 3D surface extrapolation from sparse data Medical Image Analysis 2007 11 99 109 10.1016/j.media.2006.05.001 17349939

[B11] Schmutz B Reynolds KJ Slavotinek JP Development and validation of a generic 3D model of the distal femur Computer Methods in Biomechanics and Biomedical Engineering 2006 5 305 312 10.1080/10255840600935217 17132616

[B12] Zachow S Zilske M Hege HC 3D reconstruction of individual anatomy from medical image data: segmentation and geometry processing Proceedings of the CADFEM Users Meeting 2007 Dresden, Germany

[B13] Yoo T Ed Insight into Images 2004 AK Peters

[B14] Yushkevich PA Piven J Hazlett HC Smith RG Ho S Gee JC Gerig G User-guided 3D active contour segmentation of anatomical structures: Significantly improved efficiency and reliability Neuroimage 2006 3 1116 1128 10.1016/j.neuroimage.2006.01.015 16545965

[B15] Chen T Metaxas D A hybrid framework for 3D medical image segmentation Medical Image Analysis 2005 6 547 565 10.1016/j.media.2005.04.004 15896997

[B16] Xiao M Soh J Meruvia-Pastor O Osborn D Lam N Hallgrímsson B Sensen CW An efficient virtual dissection tool to create generic models for anatomical atlases Studies in Health Technology and Informatics 2009 142 426 428 19377199

[B17] Schroeder W Martin K Lorensen B The Visualization Toolkit 2006 Prentice-Hall

[B18] Rasband WS ImageJ. U. S. National Institutes of Health, Bethesda, Maryland, USA 1997 http://rsb.info.nih.gov/ij/

[B19] Kristensen E Parsons TE Hallgrímsson B Boyd SK A novel 3D image-based morphological method for phenotypic analysis IEEE Transactions on Biomedical Engineering 2008 12 2826 2831 10.1109/TBME.2008.923106 19126464

[B20] Dice LR Measures of the amount of ecologic association between species Ecology 1945 26 297 302 10.2307/1932409

[B21] Guimond A Meunier J Thirion PJ Average brain models: a convergence study Computer Vision and Image Understanding 2000 2 192 210 10.1006/cviu.1999.0815

[B22] Guimond A Meunier J Thirion JP Automatic computation of average brain models Lecture Notes in Computer Science: Medical Image Computing and Computer-Assisted Intervention 1998-MICCAI'98 1998 1496 Berlin Heidelberg: Springer 631 640

[B23] Schaefer S Warren J Dual marching cubes: primal contouring of dual grids Proceedings of the 12th Pacific Conference on Computer Graphics and Applications: October 2004 2004 Seoul, Korea 70 76 full_text

[B24] Schaefer S Ju T Warren J Manifold dual Contouring IEEE Transactions on Visualization and Computer Graphics 2007 3 610 619 10.1109/TVCG.2007.1012 17356225

[B25] Bayly PV Black EE Pedersen RC Leister EP Genin GM In vivo imaging of rapid deformation and strain in an animal model of traumatic brain injury Journal of Biomechanics 2006 6 1086 1095 10.1016/j.jbiomech.2005.02.014 PMC1479313 16549098

[B26] Sensen CW Using CAVE^® ^technology for functional genomics studies Diabetes Technology & Therapeutics 2002 4 867 871 10.1089/152091502321118874 12614491

